# Systemic Sodium Iodate Injection as a Model for Expanding Geographic Atrophy

**DOI:** 10.1167/tvst.14.1.9

**Published:** 2025-01-10

**Authors:** Brandon D. Anderson, Brent A. Bell, Ying Song, Timothy T. Lee, Tan Wang, Joshua L. Dunaief

**Affiliations:** 1 FM Kirby Center for Molecular Ophthalmology, Scheie Eye Institute, Department of Ophthalmology, University of Pennsylvania Perelman School of Medicine, Philadelphia, PA, USA; 2 Department of Ophthalmology, Peking Union Medical College Hospital, Chinese Academy of Medical Sciences & Peking Union Medical College, Beijing, China

**Keywords:** sodium iodate, oxidative stress, geographic atrophy, retinal degeneration, mouse model

## Abstract

**Purpose:**

Geographic atrophy (GA), an advanced form of dry age-related macular degeneration (AMD), has limited treatment options. This study introduces a novel mouse model featuring an expanding GA patch that can be used to test mechanisms and therapeutics.

**Methods:**

C57Bl/6J male mice (n = 96) aged 9–10 weeks received an intraperitoneal (IP) injection of 20 mg/kg sodium iodate (NaIO_3_). In vivo confocal scanning laser ophthalmoscope (cSLO) and optical coherence tomography imaging were done at one, four, eight, and 16 weeks after injection, with GA area measurements taken at weeks 8 and 16. Mice were euthanized on weeks 8 and 16 for histological analysis.

**Results:**

Administration of 20 mg/kg intraperitoneal NaIO_3_ caused variable damage levels. Approximately 22% of cases showed damage (speckled autofluorescence) covering 35% to 90% of the 102° field of view cSLO image at one week after injection. These mice developed an expanding patch of GA by week 8, with a mean 1.45-fold increase in area by week 16. This region showed complete photoreceptor and retinal pigment epithelium loss and complement activation at the atrophy edge, whereas the inner retina remained undamaged. Mice with less damage (48% of cases) only developed incomplete outer retinal degeneration, and mice with more damage (30% of cases) had too much GA for measurable expansion.

**Conclusions:**

Although expanding GA formed in only 22% of mice, the model's simplicity and predictability for GA development via one-week post-injection imaging make it suitable for GA therapeutic experimentation.

**Translational Relevance:**

This murine model provides a valuable tool for testing GA therapies, mirroring clinical endpoints relevant to human trials.

## Introduction

Geographic atrophy (GA) is an advanced form of age-related macular degeneration (AMD), a leading cause of irreversible vision loss among the elderly worldwide. Its hallmark features include progressive degeneration of the retinal pigment epithelium (RPE) and photoreceptors, likely driven by oxidative stress and chronic inflammation.^1^^,^^2^ GA is characterized by distinct regions of RPE and outer retinal atrophy, often referred to as “geographic” because of their focal patterns and distribution across the macula. The severity of vision loss varies depending on the location of these lesions, with foveal involvement leading to loss of central vision essential for tasks like reading and driving. In contrast to neovascular AMD, the other advanced form of AMD, therapeutic options for GA are relatively recent, with the introduction of the first two drugs approved by the Food and Drug Administration in 2023. Pegcetacoplan, a monthly intravitreally-injected C3 inhibitor, exhibits a 21% reduction in GA growth in the initial year,^3^ whereas avacincaptad pegol, a monthly intravitreally-injected C5 inhibitor, demonstrates a 27% decrease in GA growth over the same period.^4^ Additional research building on the initial success of these advancements is needed. Effective models of GA are essential for the development of therapeutic interventions aimed at slowing or halting disease progression and preserving visual function in affected individuals.

An effective model of GA must have several key components. It should feature an area of complete RPE and outer retinal atrophy (cRORA), encircled by a zone of incomplete RPE and outer retinal atrophy (iRORA).^5^ Additionally, these models should demonstrate chronic enlargement of the atrophic area. Similar to clinical trials, the efficacy of therapies should be evaluated based on their ability to decelerate the rate of GA expansion,^6^ so researchers need to be able to precisely measure GA growth in the model. Although the mechanisms underlying human GA are not understood, an ideal model would also exhibit molecular hallmarks of putative GA drivers such as complement activation, microglia and macrophage recruitment, and oxidative stress.^2^^,^^7^

Several models of GA have been developed, including laser-induced GA,^8^^,^^9^ subretinal injection of sodium iodate (NaIO_3_),^10^^–^^12^ and intravitreal injection of iron.^13^ These models demonstrate areas of outer retinal atrophy that progressively expand over several months. Despite these advancements, therapeutic experiments using these models have yet to be published. Although numerous other models of photoreceptor and RPE degeneration have been established, only a few present distinct regions of complete outer retinal atrophy characteristic of GA.^14^

NaIO_3_ is used as a model of oxidative stress in the retina because of its ability to selectively damage the RPE and photoreceptors when administered systemically, either intravenously (IV) or intraperitoneally (IP). In therapeutic studies, it is typically administered at a dose of 40 mg/kg with an endpoint of one week post-injection. At this dose and timepoint, widespread atrophy of photoreceptors and RPE occurs, resulting in an area of cRORA or iRORA covering most of the retina. Although this model aids in understanding the therapeutic potential of compounds against oxidative stress-induced retinal degeneration, it does not replicate the disease stage targeted by most GA therapies. This standard NaIO_3_ method does not induce small, geographic regions of atrophy that subsequently expand over time.

To create smaller, more geographic regions of damage with NaIO_3_, subretinal injections have been used instead of IP or IV administration.^10^^–^^12^ Expansion of a cRORA region over several months has been demonstrated using this method.^12^ Lower doses administered via IP (20 mg/kg) or IV (<15 mg/kg) injections can also induce smaller areas of atrophy more akin to GA, with the added benefits of easier administration and symmetrical damage in both eyes.^15^^–^^17^ However, at the typically studied one-week timepoint of these low-dose systemic injections, the outer nuclear layer (ONL), although thinned, remains present. Longer timepoints have not been studied with these doses, leaving it uncertain whether they can model GA as effectively as subretinal NaIO_3_ injections in terms of cRORA development and expansion. Additionally, the high variability in induced damage with these lower systemic doses needs to be better understood before they can be reliably used as a model for therapeutic testing.^15^

The purpose of this study is to present a mouse model with morphological features GA, induced by an IP injection of 20 mg/kg NaIO_3_. This single-injection model induces GA-like features within eight weeks after injection, with observable expansion of atrophic lesions by 16 weeks. The GA development occurs only in a subset of mice injected at this dose, so this study also establishes predictive parameters measured one week after injection to anticipate which mice will develop GA. This model presents researchers with a new tool for preclinical investigations, offering a new platform to explore GA mechanisms and novel therapeutics and propel advancements aimed at improving patient outcomes.

Although this mouse model replicates certain morphological features associated with GA, human GA is a complex disease. Within the present study, when “GA” is referenced in the context of the mouse retina, it is specifically describing an area characterized by expanding cRORA.

## Material and Methods

### Animals

C57Bl/6J male mice were acquired from Jackson Laboratories (strain number 000664; Bar Harbor, ME, USA) at eight weeks of age. Male mice were used for this initial IP NaIO_3_ GA study to minimize variability, especially considering the variability at the low dose of 20 mg/kg and the sex differences in the NaIO_3_ model.^15^ C57Bl/6J mice do not have the Rd8 mutation in the *Crb1* gene.^18^ They were kept at a 12-:12-hour light/dark cycle and given a standard laboratory diet as desired. The average illuminance, measured from 11 locations 1 m above the floor, was 1262 ± 392 lx (ILT1400 Lux Meter; International Light Technologies, Peabody, MA, USA). All experiments were approved by the Institutional Animal Care and Use Committee of the University of Pennsylvania (Philadelphia, PA, USA) and were performed in accordance with the ARVO Statement for the Use of Animals in Ophthalmic and Vision Research. A total of 96 mice were used.

### NaIO_3_ Administration

Mice were nine to 10 weeks old at the time of injection. NaIO_3_ (Sigma-Aldrich, St. Louis, MO, USA) was prepared by dissolving it in saline solution (0.9% sodium chloride injection USP; B. Braun, Melsungen, Germany) at a concentration of 1% (w/v). This was injected intraperitoneally at 2 µL/g to get a dose of 20 mg/kg. A 31-gauge insulin syringe with an 8 mm length and 300 µL capacity (BD Bioscience, Franklin Lakes, NJ, USA) was used. Once the mouse was scruffed and the tail secured, the injection was administered on the right side of the abdomen, in the middle of where the two right abdominal nipples would typically appear in female mice. The needle was inserted at an approximate 45° angle from the abdomen and was inserted approximately 75% of its length.

### In Vivo Imaging

One, four, eight, and 16 weeks after NaIO_3_ was administered, in vivo retinal images of both eyes were taken using a Spectralis HRA confocal scanning laser ophthalmoscope (cSLO; Heidelberg Engineering, Franklin, MA, USA) and a Bioptigen Envisu R2200 ultra-high resolution spectral-domain optical coherence tomography system (SD-OCT or OCT; Leica Microsystems, Deerfield, IL, USA).

Mice were prepared for in vivo imaging by applying a single drop of a 2:1 blend of 1% tropicamide and 2.5% phenylephrine (Akorn, Lake Forest, IL, USA) to each eye several minutes before anesthesia. Anesthesia was induced through an IP injection of ketamine (93–98 mg/kg, Dechra Veterinary Products, Overland Park, KS, USA) and xylazine (10–11 mg/kg; Akorn). Topical anesthesia with 1% tetracaine (Alcon Laboratories, Geneva, Switzerland) was then applied to both corneas, followed by the application of Refresh Artificial Tears (Allergan, Irvine, CA, USA) and protective eye shields to prevent corneal desiccation and media opacity.^19^ The mice were then transferred to the cSLO imaging platform and imaged before proceeding to SD-OCT imaging.

The cSLO images were captured using the blue autofluorescence (BAF) channel (486 nm raster scanned laser for excitation and a 500–680 nm bandpass range for emission collection) and the infrared autofluorescence (IRAF) channel (795 nm raster scanned laser for excitation and an 800 nm longpass filter for emission collection). These images were acquired using the 102° Ultrawide Field lens, affording a ∼3.4 mm field of view (FOV) of the mouse fundus. System focus was adjusted to target the RPE, with images capturing the optic nerve centrally positioned within the image FOV, as described previously.^20^ Twenty-five frames were acquired in high-speed mode (768 × 768 pixels) from each mouse retina, and real-time co-registration and averaging were performed using the Heidelberg Eye Explorer (HEYEX 1) software's automatic real-time processing feature. Averaged images were auto-normalized to optimize contrast.

After completing cSLO imaging, SD-OCT images were acquired using a 45° FOV lens (∼1.4 mm B-scan width), with the optic nerve positioned centrally. Orthogonal B-scans (1400 A-scans/2 B-scans × 15 frames/B-scan) were taken at 0° and 90° to capture horizontal and vertical images. Peripheral B-scans were performed in the temporal, nasal, superior, and inferior regions of both eyes, with the optic nerve positioned on one side for more comprehensive coverage.

When the imaging process was done, mice were given an IP injection of 1.5 mg/kg atipamezole HCL (Modern Veterinary Therapeutics, Miami, FL, USA) to aid anesthesia recovery. To protect the cornea during recovery, Puralube Vet Ointment (Dechra Veterinary Products) was applied to cover the eyes.

### In Vivo Image Analysis

The 15 SD-OCT B-scan image frames from each orthogonal B-scan were co-registered and averaged in ImageJ (NIH, Bethesda, MD, USA) using the OCT Volume Averager plugin^21^^,^^22^ and a custom-made python script. To show where these images were in relation to the cSLO images, the *en face* Volume Intensity Projection images taken by the OCT imager were overlaid on the cSLO images in Photoshop (Adobe, San Jose, CA, USA) at 50% opacity, and the vasculature was aligned. The vertical center of the Volume Intensity Projection OCT images, where the B-scans were located, was marked on the cSLO images accordingly.

In cSLO images taken one week after injection, the speckled autofluorescent regions representative of damage were quantified. In cSLO images captured eight and 16 weeks after injection, the sharply demarcated dark regions indicative of GA were quantified. The GA regions in the eight-week images were less clearly defined, and regions were identified by discerning distinct lines of dark areas adjacent to lighter regions. The quantification for all timepoints was done in Photoshop through the following steps: (1) the image layer was duplicated, and the art history brush was applied to this duplicate step. (2) Everything in the duplicated layer was deleted by selecting all and clicking “delete.” (3) Using the art history brush, the damaged region was carefully restored onto the deleted layer. To facilitate identification, a green border was added around the damaged region layer by adding a 2-pixel stroke using the layer style dialog box. (4) An ellipse was created around the eye to delete any areas outside of the eye. (5) The pixel count of the damaged region layer was recorded using the histogram panel. The percentage of damaged area was calculated by dividing the damaged pixel count by the total pixel count of the eye. All GA regions in the eight- and 16-week images underwent verification by a second evaluator.

### Histology

Sixteen weeks after NaIO_3_ injection, mice were euthanized by CO_2_ asphyxiation followed by cervical dislocation. Their eyes were enucleated while preserving the third eyelid and placed in a solution of 2% paraformaldehyde and 2% glutaraldehyde (diluted from 16% paraformaldehyde and 8% glutaraldehyde; Electron Microscopy Sciences, Hatfield, PA, USA) in 1x phosphate-buffered saline solution, and stored at 4°C. Later, the eyes were dissected to remove the cornea, iris, and lens, leaving behind the “eye cup,” which was then dehydrated through two 15-minute washes each of 75% EtOH (Decon Labs, King of Prussia, PA, USA), 95% EtOH, and 100% EtOH. The eye cups were then put in an infiltration solution (JB-4 Plus Embedding Kit; Polysciences, Warrington, PA, USA) overnight. The following day, the eyes were embedded using a glycol methacrylate-based plastic resin embedding medium (JB-4 Plus Embedding Kit; Polysciences), ensuring orientation to encompass both superior and inferior regions in the resulting sections.

Using a rotary microtome (Leica RM2165; Leica Biosystems, Wetzlar, Germany), 3 µm sections were obtained. Sections containing both the optic nerve and an area of GA were stained with toluidine blue (Sigma-Aldrich) for 10 seconds, followed by washing with water. The slides were rinsed under running water for two minutes, then in still water for five minutes. After drying, the slides underwent a series of ethanol and xylene washes: 95% ethanol for one minute, 95% ethanol for 30 seconds, 100% ethanol for one minute, 100% ethanol for 30 seconds, xylenes (Histoprep; ThermoFisher Scientific, Waltham, MA, USA) for two minutes, and xylenes for one minute. Permount mounting medium (ThermoFisher Scientific) was applied immediately after the last xylenes wash, and a coverslip was added.

Images at magnification ×20 were captured using an Aperio ScanScope AT Turbo (Aperior, Vista, CA, USA). The images’ levels and color were adjusted in Photoshop.

### Immunohistochemistry

For cross-sectional images, eight weeks after NaIO_3_ injection, mice were euthanized using CO_2_ asphyxiation followed by cervical dislocation. The eyes, with the third eyelid preserved, were enucleated and placed in a 4% paraformaldehyde 1x phosphate-buffered saline solution (diluted from 16% stock; Electron Microscopy Sciences, Hatfield, PA, USA) for 15 minutes. Subsequently, the cornea, iris, and lens were dissected, and the remaining eye tissue was dehydrated overnight in a 30% sucrose solution. The eyes were then embedded in Tissue-Tek O.C.T. compound (Sakura Finetek, Torrance, CA, USA), flash-frozen, and sectioned into 10 µm-thick slices on the sagittal plane using a Leica CM3050 S cryostat (Leica Biosystems) to capture both the superior GA region and the inferior normal region.

Cross-sections were selected to include the GA region and the optic disc. These sections underwent permeabilization and blocking for 1 hour at room temperature in tris buffered saline solution containing 0.1% Triton X-100 (Sigma-Aldrich) (TBST) with 10% normal donkey serum (NDS; Jackson ImmunoResearch, West Grove, PA). After this, sections were incubated overnight at 4°C with a primary antibody (Table, rows 1–3) diluted in TBST and 2% NDS. Control sections were treated identically, with the exception of omitting the primary antibody. After the primary antibody incubation, sections were washed three times with TBST and then incubated with a secondary antibody (Table, rows 5–7) diluted in TBST and 2% NDS for one hour at room temperature, followed by more washes. Nuclei were counterstained with DAPI Fluoromount-G (SouthernBiotech, Birmingham, AL, USA). Imaging of sections was conducted using epifluorescence microscopy with the Nikon Eclipse 80i microscope and DS-QiMc camera (Nikon, Tokyo, Japan), using Nikon Elements software. Identical exposure parameters were maintained for each image within a given antibody.

**Table.** List of Primary and Secondary Antibodies Used

	Antibody	Source	Identifier	Dilution
1	Goat anti-C3	MP Biomedicals	0855474	1:200
2	Rat anti-C3b/iC3b/C3c	Hycult Biotech	HM1078	1:50
3	Chicken anti-GFAP	Abcam	ab4674	1:100
4	Rabbit anti-ZO-1	Invitrogen	40-2200	1:200
5	Alexa Fluor 647 donkey anti-goat	Jackson ImmunoResearch	705-605-147	1:200
6	Cy3 donkey anti-rat	Jackson ImmunoResearch	712-165-153	1:200
7	Cy3 donkey anti-chicken	Jackson ImmunoResearch	703-165-155	1:200
8	Alexa Fluor 647 donkey anti-rabbit	Jackson ImmunoResearch	711-605-152	1:200

Primary (rows 1–4) and secondary (rows 5–8) antibodies used for immunohistochemistry, suppliers, catalog numbers, and dilutions.

For RPE flatmounts, after enucleation, eyes were fixed in 4% paraformaldehyde for eight minutes. The cornea, lens, iris, and retina were then dissected out. The remaining eye cup was then blocked with 10% NDS in TBST for 30 minutes. They were then incubated in primary antibody (Table, row 4) diluted in TBST and 2% NDS overnight at 4°. The next day, the eye cups were washed with TBST three times. Incubation with the secondary antibody (Table, row 8) in 2% NDS and TBST was done for two hours at room temperature, followed by three washes of TBST and one wash of TBS. The eye cup was then placed on a slide, and radial incisions were made so the eye would lay flat on the slide. Imaging was done similar to the cross-section imaging described above, and images were stitched together using Photoshop's Photomerge function.

### Statistics

Statistical analyses were done in Prism 10.1.1 (GraphPad Software, Boston, MA, USA). One eye was excluded from the GA expansion calculations because of insufficient clarity in the cSLO image at eight weeks, precluding accurate quantification.

Descriptive statistics are presented as mean ± standard error of the mean (SEM), with significance denoted by asterisks (*P < 0.05, **P < 0.01, ***P < 0.001, ****P < 0.0001). The significance of GA expansion was assessed using a one-sample *t*-test, comparing the fold change of expansion to a theoretical value of 1. Correlation between damage area at one week and GA formation was evaluated using an independent two-sample *t*-test. Two-tailed *P* values were determined for both one-sample and two-sample *t*-tests.

XY plots in the supplemental figures were subjected to simple linear regression analysis, wherein the null hypothesis of a slope of 0 (indicating no relationship between the x and y variables) was tested. Additionally, the 95% confidence bands surrounding the best-fit line were reported. To evaluate the relationship between eight-week atrophy area and its expansion shown in these graphs, each fold change value was transformed by taking its square root. However, because this transformation did not eliminate the observed effect, the original fold change values rather than the transformed ones were used.

## Results

### Geographic Atrophy Formation in Superior Region of Retina

**Figure 1.**
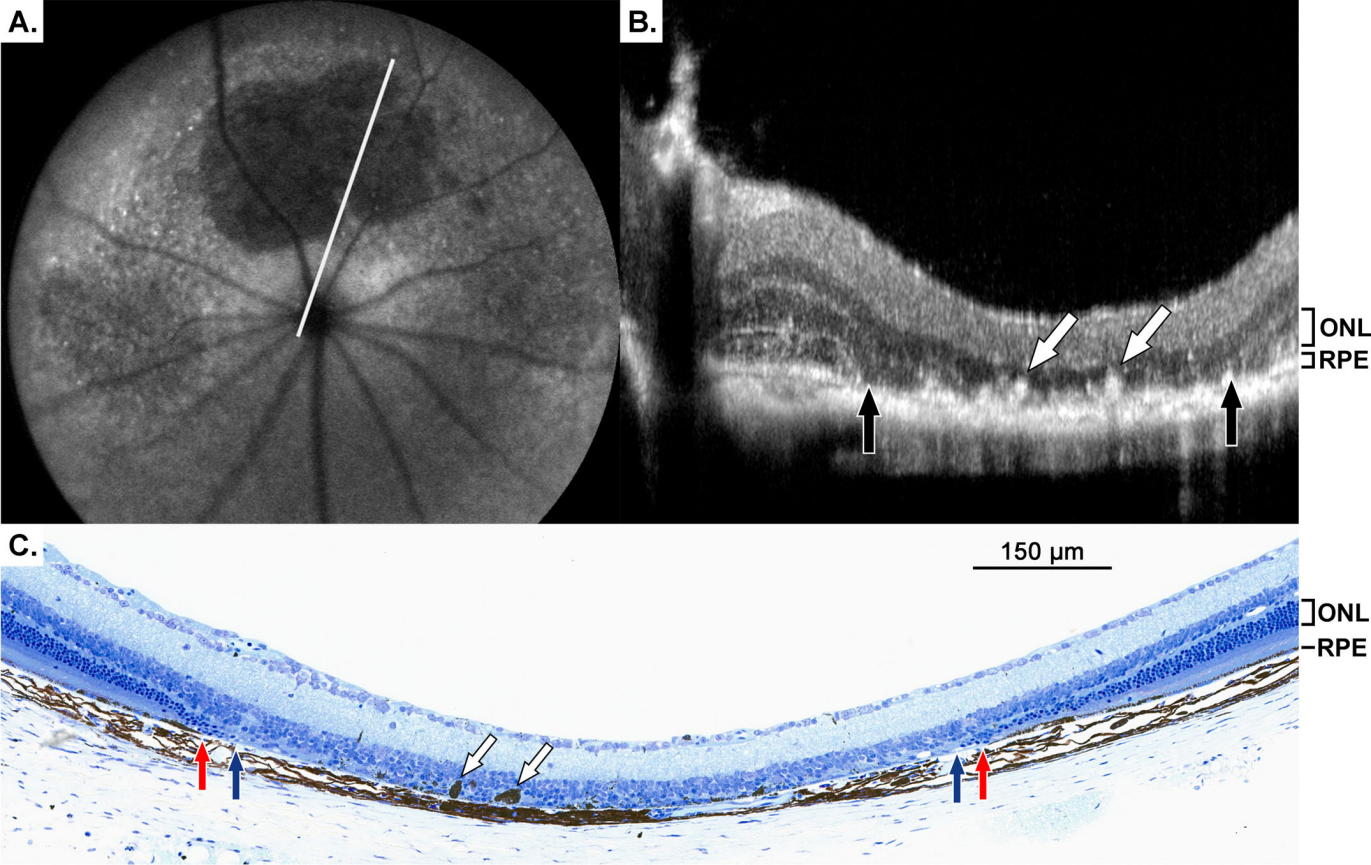
Geographic-atrophy-like phenotype 16 weeks after injection. (**A**) cSLO and (**B**) OCT images taken 16 weeks after injection of 20 mg/kg NaIO_3_. The *white line* in the cSLO image corresponds to the OCT line scan in panel B. (**B**) *Black arrows* represent cRORA borders and *white arrows* show IHRF. (**C**) Plastic section of the GA region stained with toluidine blue. *Scale bar*: 150 µm. *Red arrows* show where the RPE layer ends, *blue arrows* show where photoreceptor nuclei end, and *white arrows* show pigmented cells that correspond to the IHRF found in the OCT images. Images from other mice can be found in Supplementary Fig. S1.

Male mice age nine to 10 weeks injected with 20 mg/kg NaIO_3_ developed well-defined GA 16 weeks after the injection (Fig. 1; additional examples in Supplementary Fig. S1). These GA regions formed predominantly in the superior region of the retina, presenting as markedly hypoautofluorescent areas in cSLO BAF images (Fig. 1A). Surrounding these areas, the cSLO images showed a less hypoautofluorescent region, indicating partial damage. RPE flatmounts revealed enlarged, nonhexagonal RPE cells in this partially damaged zone, whereas no RPE remained in the most hypoautofluorescent area (Supplementary Fig. S2).

In OCT images, these GA regions showed complete degeneration of the ONL (Fig. 1B). Within this cRORA area were intraretinal hyperreflective foci (IHRF; white arrows in Fig. 1B). Further from the GA lesion, the ONL thickness was normal but tapered rapidly as the line scan neared the lesion.

These regions of GA lacked both photoreceptors and RPE, whereas the inner retina remained intact (Fig. 1C). This region of cRORA was encircled by a zone of iRORA. Within this iRORA area, the ONL displayed thinning, yet remained partially intact. At the transition area surrounding the cRORA, RPE degeneration had progressed to the extent that photoreceptors were present without RPE support beneath them (Fig. 1C area between red and blue arrows).

### Prediction and Expansion of Atrophy Area

**Figure 2.**
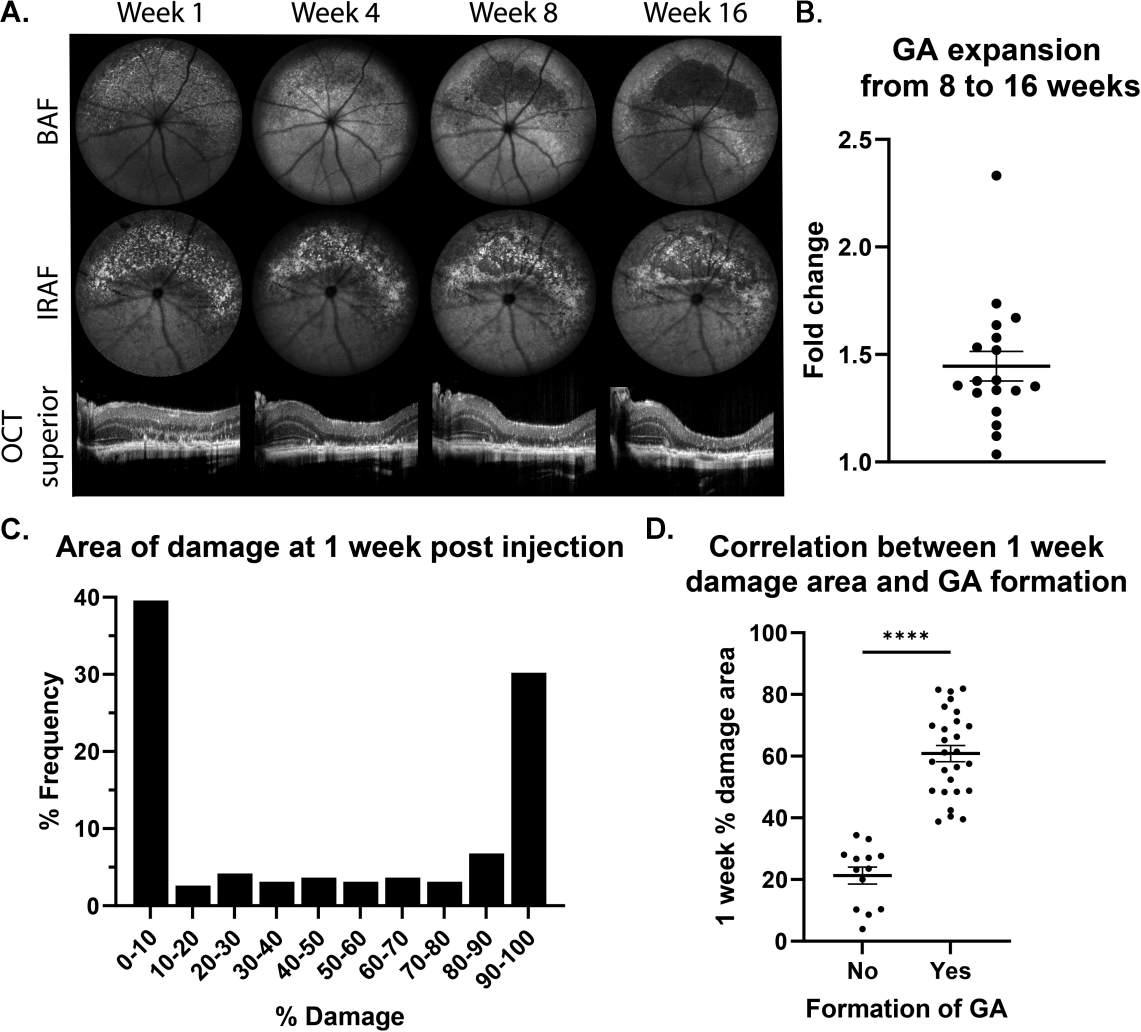
Prediction and expansion of GA. (**A**) Example images from one eye taken at one, four, eight, and 16 weeks after the NaIO_3_ injection. OCT images from the superior retina were taken with the optic nerve on the left side; the right side of the image is superior to the optic nerve. (**B**) Expansion of the GA from eight to 16 weeks, reported as fold change (n = 18). The mean is statistically different from 1 (*P* < 0.0001, one-sample *t* test). (**C**) Variability of damage seen one week after injection of 20 mg/kg NaIO_3_ (n = 192 eyes). Damage is quantified as the percent of the retina showing speckled autofluorescence in BAF images captured with a 102° FOV lens. (**D**) Correlation between one-week post-injection damage area and formation of cRORA by eight weeks (n = 40). Each *dot* represents one eye.

A region of cRORA was present as early as eight weeks after the injection of 20 mg/kg NaIO_3_ (Fig. 2A). Over the subsequent eight weeks, this region progressively darkened and became more defined. Similar to human patients with GA, the area affected by GA also expanded (Fig. 2A, see Supplementary Fig. S3A for other examples). By 16 weeks after injection, on average the GA region had grown to 1.45 times its size at eight weeks (Fig. 2B). The area and growth of atrophy was similar between the OD/OS eyes of each mouse (Supplementary Fig. S3B, S3C). Smaller areas of GA (measured at eight weeks) had a greater fold change of expansion from eight to 16 weeks after injection (Supplementary Fig. S3D), even after applying a square root transformation to the fold change, a method commonly used in clinical studies to mitigate the confounding effect of baseline size on the rate of enlargement.^23^

Although cRORA formation took up to eight weeks, initial damage induced by NaIO_3_ developed quickly and was observable within a week after injection in cSLO images (as speckled hypo/hyperautofluroescence) and OCT images (as thinned but not completely degenerated ONL) (Fig. 2A, first column). The damage at this point is only iRORA. By four weeks after injection, OCT images revealed complete loss of the ONL layer, although this cRORA region was less defined in cSLO images (Fig. 2A, second column). The cRORA area at 16 weeks never extended beyond the initial iRORA damage area observed at one week (Supplementary Fig. S4).

Not all mice injected with 20 mg/kg IP NaIO_3_ developed GA. Most mice either showed near-complete damage or no discernible damage at all (Fig. 2C). The formation of cRORA could be predicted by the area of the damage one week after injection. If the damage (seen as speckled BAF in cSLO images) covered more than 35% of the 102° FOV image, GA formation was anticipated (Fig. 2D). In contrast, damage below this threshold (48% of mice injected) did not lead to cRORA (Supplementary Fig. S5A). Eyes with an excessive area of damage (over 90% speckled BAF at one week; 30% of mice injected) had poorly defined cRORA borders by eight weeks in the cSLO images. In these eyes, the 102° FOV area was already dominated by cRORA, making any further atrophy expansion unmeasurable (Supplementary Fig. S5B). Eyes with 35% to 90% damage at one week (22% of mice injected) had sufficient damage to develop cRORA by eight weeks, but not so much that there wasn't room to measure expansion between eight and 16 weeks after injection.

### Complement Activation Along the Border of Atrophy

**Figure 3.**
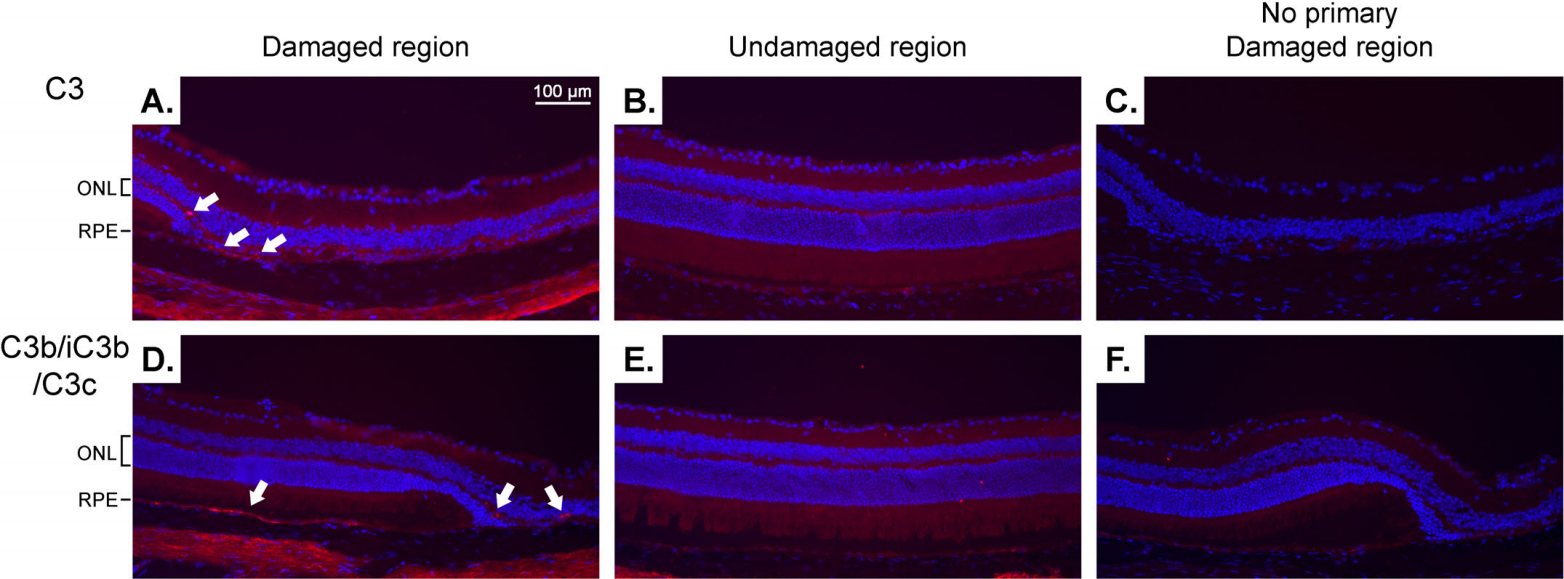
Immunohistochemistry imaging of complement proteins. Representative images of (**A**–**C**) C3 (*red*) and (**D**–**F**) C3b/iC3b/C3c (*red*) at (**A**, **C**, **D**, **F**) the border of atrophy and (**B**, **E**) the undamaged region of the same retina. (**A**, **C**) cRORA occupies most of the right side of the image with some iRORA on the left side. (**D**, **F**) cRORA is located at the far right side, the iRORA region is more medial, and an undamaged region is on the left side. *White arrows* indicate fluorescent puncta indicative of (**A**) C3 and (**D**) C3b/iC3b/C3c in the OPL and RPE layers. *Scale bar in A*: 100 µm, applies to all images. Nuclei are labeled with DAPI (*blue*).

To further compare the NaIO_3_ mouse model of GA to human GA, immunohistochemistry was performed on mice eight weeks after injection with 20 mg/kg NaIO_3_, a timepoint when atrophy is established and continues to spread. Complement component 3 (C3), a central component of the complement system and a target of pegcetacoplan, was present at the edge of the iRORA region in the outer plexiform layer (OPL) and at the edge of the cRORA region in the RPE layer (Fig. 3A), suggesting activation of the complement system. This C3 labeling was not observed in the undamaged regions of the retinas (Fig. 3B) nor in sections without the C3 primary antibody (Fig. 3C).

Further progression of the complement activation pathway was indicated by the presence of C3’s cleaved products: C3b, iC3b, and/or C3c (Fig. 3D). These cleaved products were present in the RPE layer in the undamaged region surrounding the atrophy region as well as in the OPL of the cRORA and iRORA regions (Fig. 3D, white arrows). The undamaged regions farther from the atrophy did not have these products (Fig. 3E), and these fluorescent puncta were not seen without the primary antibody (Fig. 3F).

## Discussion

Injecting NaIO_3_ intraperitoneally at a dose of 20 mg/kg can induce an expanding degeneration with morphological features of GA, offering researchers a new platform for investigating mechanisms and potential therapeutics for this disease. GA profoundly impacts vision and has affected numerous individuals. Despite the recent approval by the Food and Drug Administration of two novel therapeutics for GA, further research is essential to uncover and enhance treatment options for affected patients.

### Comparison of This Model to GA and Other GA Models

This model includes several key features of GA: (1) There was a progressive expansion of the cRORA region over time. In this model, the cRORA region expanded on average 1.45 times from eight to 16 weeks after injection. The eight-week post-injection timepoint was the earliest suitable time for assessing dark region expansion in BAF images using a cSLO system, because earlier timepoints lacked defined borders for accurate quantification. (2) The iRORA region immediately surrounding the cRORA exhibited RPE death extending beneath the thinning ONL.^24^^,^^25^ (3) OCT images had IHRF present. In human patients, the occurrence of IHRF indicates a poor prognosis for GA progression.^26^ It is believed that IHRF represent migration of RPE cells to inner retinal layers.^27^ In this model, plastic sections confirmed the observed IHRF as pigmented cells that could be RPE or macrophages/microglia. (4) Complement C3 and its cleaved products were found at the border of atrophy. C3 activity has not only been noted at the edge of human GA,^28^ but the C3 inhibitor pegcetacoplan has also been shown to slow the progression of atrophy in human GA.^3^ (5) Müller cell activation, indicated by glial fibrillary acidic protein (GFAP) expression, was observed in the atrophy region and the surrounding area (Supplementary Fig. S6). In human GA, there is a marked increase in Müller cell activation within and around the atrophic regions, evidenced by elevated GFAP and vimentin expression.^29^^,^^30^

To our knowledge, this is the first model that induces progressive regional atrophy of the outer retina through systemic drug administration. This method offers an efficient alternative, avoiding the time-intensive processes involved in developing genetic knockout models or using more complex protocols. Alternative models for GA include subretinal injection of NaIO_3_ in pigs and rats,^10^^–^^12^ laser-induced GA in mice,^8^^,^^9^ and intravitreal injection of iron in mice.^13^ Like the IP injection of NaIO_3_ detailed here, these models also require several months for development and progression. Although these models do have expansion, the expansion rates were not described, so it is unclear how they compare to the IP injection of NaIO_3_. All of these models induce regions of cRORA and leave the inner retina unaffected, except for the subretinal injection of NaIO_3_, where instances of inner retinal degeneration did occur.^12^

### Further Potential Optimization of This Model

Optimization of this model should focus on increasing the proportion of mice that develop expanding GA. In this study, only 22% of the 96 mice showed moderate damage that could expand, whereas 48% had insufficient damage to develop cRORA, and 30% experienced excessive damage, leaving no room for expansion. Increasing the dose may boost the number of mice with the expanding cRORA phenotype. Previous tests showed that 25 mg/kg IP causes excessive damage,^15^ but a dose in the 21–24 mg/kg range might shift more of the 48% with insufficient damage into the desired damage range.

Because a large number of mice are needed to assess the effect of sex on GA formation, only male mice were tested in this study. Our previous work suggested that female mice given 20 mg/kg IP NaIO_3_ showed a similar damage area to males, though the sample size was too small for definitive conclusions.^15^ Other studies using IV NaIO_3_ at lower doses (10–11 mg/kg) found that females had larger damage areas than males.^16^ Although these findings have implications for the 20 mg/kg IP dose, further research is needed to determine whether females would have a higher proportion developing ideal damage for the expanding GA model.

This model has a relatively long timeframe: GA becomes visibly detectable via cSLO imaging at two months after injection, with an additional two months needed for significant growth. This is comparable to other GA models and GA progression in humans is even longer. However, cRORA was seen as early as four weeks after injection in OCT images. The cSLO images were used for their ease in measuring atrophy, but if a reliable method for measuring the GA area in OCT images were developed, the experimental timeline could be shortened.

### Suggestions for Use of This Model

Predicting GA formation by one week after injection allows researchers to decide whether additional mice should be injected with NaIO_3_ to ensure the study is sufficiently powered. To quantify damage, it is recommended to use a cSLO imaging system to generate BAF images with a 102° FOV lens. Mice should have more than 35% of the 102° image damaged (speckled autofluorescence) at the one-week timepoint to develop cRORA by eight weeks.

IP injection was selected for this study because of its ease of use, the symmetry of damage between the eyes, and prior optimization.^15^ However, alternative methods for NaIO_3_ dosing are available. If IV injections, which also induce symmetrical GA damage, are used, dosing optimization would be necessary because of the smaller amounts of NaIO_3_ required to achieve comparable results.

Although AMD is an age-related disease, previous work has shown that using older mice (18–24 months old) in the NaIO_3_ model leads to more variability and damage patterns likely unsuited for GA expansion studies.^15^ Young mice (around two months old) are recommended instead.

Researchers conducting therapeutic studies using this model should not administer the therapeutic before NaIO_3_ administration. Although that is common in many oxidative stress studies, it is likely to prevent the formation of GA. Instead, it is recommended to allow the initial GA to form by eight weeks after NaIO_3_ injection before administering the therapeutic. This ensures that the study endpoint focuses on GA expansion rather than GA formation. If therapeutics with longer onset times, such as adeno-associated viruses, are used, they should be administered at a timepoint that ensures protection is in effect by eight weeks after NaIO_3_-injection.

## Conclusions

The use of 20 mg/kg IP NaIO_3_ to induce GA in mouse models offers a valuable tool for investigating potential therapeutics for this debilitating retinal condition. Despite some limitations, such as variability in GA induction and the time required for its development, this model closely mirrors key aspects of human GA pathology, including the progressive expansion of the atrophic region over time. Researchers can leverage this model to explore novel treatments and optimize dosing strategies to effectively mitigate GA progression. Continued efforts to refine experimental protocols and test for mechanistic similarities to human GA will further enhance the utility of this model in preclinical research, ultimately leading to improved clinical outcomes for individuals with GA.

## Supplementary Material

Supplement 1

## References

[1] Abidi M, Karrer E, Csaky K, Handa JT. A Clinical and Preclinical Assessment of Clinical Trials for Dry Age-Related Macular Degeneration. *Ophthalmol Sci*. 2022; 2(4): 100213. 36570624 10.1016/j.xops.2022.100213 PMC9767821

[2] Boyer DS, Schmidt-Erfurth U, van Lookeren Campagne M, Henry EC, Brittain C. The pathophysiology of geographic atrophy secondary to age-related macular degeneration and the complement pathway as a therapeutic target. *Retina*. 2017; 37: 819–835. 27902638 10.1097/IAE.0000000000001392 PMC5424580

[3] Heier JS, Lad EM, Holz FG, et al. Pegcetacoplan for the treatment of geographic atrophy secondary to age-related macular degeneration (OAKS and DERBY): two multicentre, randomised, double-masked, sham-controlled, phase 3 trials. *Lancet*. 2023; 402(10411): 1434–1448. 37865470 10.1016/S0140-6736(23)01520-9

[4] Jaffe GJ, Westby K, Csaky KG, et al. C5 inhibitor avacincaptad pegol for geographic atrophy due to age-related macular degeneration: a randomized pivotal phase 2/3 trial. *Ophthalmology*. 2021; 128: 576–586. 32882310 10.1016/j.ophtha.2020.08.027

[5] Sadda SR, Guymer R, Holz FG, et al. Consensus definition for atrophy associated with age-related macular degeneration on OCT: Classification of Atrophy Report 3. *Ophthalmology*. 2018; 125: 537–548. 29103793 10.1016/j.ophtha.2017.09.028 PMC11366072

[6] Csaky KG, Richman EA, Ferris FL III. Report from the NEI/FDA Ophthalmic Clinical Trial Design and Endpoints Symposium. *Invest Ophthalmol Vis Sci*. 2008; 49: 479–489. 18234989 10.1167/iovs.07-1132

[7] Cherepanoff S, McMenamin P, Gillies MC, Kettle E, Sarks SH. Bruch's membrane and choroidal macrophages in early and advanced age-related macular degeneration. *Br J Ophthalmol*. 2010; 94: 918–925. 19965817 10.1136/bjo.2009.165563

[8] Ibbett P, Goverdhan SV, Pipi E, et al. A lasered mouse model of retinal degeneration displays progressive outer retinal pathology providing insights into early geographic atrophy. *Sci Rep*. 2019; 9: 7475. 31097765 10.1038/s41598-019-43906-z PMC6522499

[9] Khan AH, Soundara Pandi SP, Scott JA, et al. A laser-induced mouse model of progressive retinal degeneration with central sparing displays features of parafoveal geographic atrophy. *Sci Rep*. 2023; 13: 4194. 36918701 10.1038/s41598-023-31392-3 PMC10014848

[10] Bhutto IA, Ogura S, Baldeosingh R, McLeod DS, Lutty GA, Edwards MM. An acute injury model for the phenotypic characteristics of geographic atrophy. *Invest Ophthalmol Vis Sci*. 2018; 59(4): AMD143–AMD151. 30208410 10.1167/iovs.18-24245 PMC6133234

[11] Monés J, Leiva M, Peña T, et al. A swine model of selective geographic atrophy of outer retinal layers mimicking atrophic AMD: A phase I escalating dose of subretinal sodium iodate. *Invest Ophthalmol Vis Sci*. 2016; 57: 3974–3983. 27479813 10.1167/iovs.16-19355

[12] Naik P, Grebe R, Bhutto IA, McLeod DS, Edwards MM. Histologic and immunohistochemical characterization of GA-like pathology in the rat subretinal sodium iodate model. *Transl Vis Sci Technol*. 2024; 13(2): 10. 10.1167/tvst.13.2.10 PMC10868633 38349778

[13] Liu Y, Bell BA, Song Y, et al. Intraocular iron injection induces oxidative stress followed by elements of geographic atrophy and sympathetic ophthalmia. *Aging Cell*. 2021; 20(11): e13490. 34626070 10.1111/acel.13490 PMC8590099

[14] Soundara Pandi SP, Ratnayaka JA, Lotery AJ, Teeling JL. Progress in developing rodent models of age-related macular degeneration (AMD). *Exp Eye Res*. 2021; 203: 108404. 33340497 10.1016/j.exer.2020.108404

[15] Anderson BD, Lee TT, Bell BA, Wang T, Dunaief JL. Optimizing the sodium iodate model: Effects of dose, gender, and age. *Exp Eye Res*. 2024; 239: 109772. 38158173 10.1016/j.exer.2023.109772 PMC10922497

[16] Yang X, Rai U, Chung JY, Esumi N. Fine tuning of an oxidative stress model with sodium iodate revealed protective effect of NF-κB inhibition and sex-specific difference in susceptibility of the retinal pigment epithelium. *Antioxidants*. 2021; 11: 103. 35052607 10.3390/antiox11010103 PMC8773095

[17] Zhang N, Zhang X, Girardot PE, et al. Electrophysiologic and morphologic strain differences in a low-Dose NaIO3-induced retinal pigment epithelium damage model. *Transl Vis Sci Technol*. 2021; 10(8): 10. 10.1167/tvst.10.8.10 PMC8287050 34251426

[18] Mattapallil MJ, Wawrousek EF, Chan CC, et al. The Rd8 mutation of the Crb1 gene is present in vendor lines of C57BL/6N mice and embryonic stem cells, and confounds ocular induced mutant phenotypes. *Invest Ophthalmol Vis Sci*. 2012; 53: 2921–2927. 22447858 10.1167/iovs.12-9662 PMC3376073

[19] Bell BA, Kaul C, Hollyfield JG. A protective eye shield for prevention of media opacities during small animal ocular imaging. *Exp Eye Res*. 2014; 0: 280–287. 10.1016/j.exer.2014.01.001 PMC4173074 25245081

[20] Bell BA, Kaul C, Bonilha VL, Rayborn ME, Shadrach K, Hollyfield JG. The BALB/c mouse: effect of standard vivarium lighting on retinal pathology during aging. *Exp Eye Res*. 2015; 135: 192–205. 25895728 10.1016/j.exer.2015.04.009 PMC4446204

[21] Krebs MP, Xiao M, Sheppard K, Hicks W, Nishina PM. Bright-field imaging and optical coherence tomography of the mouse posterior eye. In: Proetzel G, Wiles MV, eds. *Mouse Models for Drug Discovery: Methods and Protocols*. Methods in Molecular Biology. Berlin: Springer; 2016: 395–415. 10.1007/978-1-4939-3661-8_20 27150100

[22] Thevenaz P, Ruttimann UE, Unser M. A pyramid approach to subpixel registration based on intensity. *IEEE Trans Image Process*. 1998; 7: 27–41. 18267377 10.1109/83.650848

[23] Yehoshua Z, Rosenfeld PJ, Gregori G, et al. Progression of geographic atrophy in age-related macular degeneration imaged with spectral domain optical coherence tomography. *Ophthalmology*. 2011; 118: 679–686. 21035861 10.1016/j.ophtha.2010.08.018 PMC3070862

[24] Bearelly S, Chau FY, Koreishi A, Stinnett SS, Izatt JA, Toth CA. Spectral domain optical coherence tomography imaging of geographic atrophy margins. *Ophthalmology*. 2009; 116: 1762–1769. 19643488 10.1016/j.ophtha.2009.04.015 PMC2738753

[25] Bird AC, Phillips RL, Hageman GS. Geographic atrophy. *JAMA Ophthalmol*. 2014; 132: 338–345. 24626824 10.1001/jamaophthalmol.2013.5799 PMC4853921

[26] Christenbury JG, Folgar FA, O'Connell R, Chiu SJ, Farsiu S, Toth CA. Progression of intermediate age-related macular degeneration with proliferation and inner retinal migration of hyperreflective foci. *Ophthalmology*. 2013; 120: 1038–1045. 23352193 10.1016/j.ophtha.2012.10.018 PMC3640702

[27] Nittala MG, Corvi F, Maram J, et al. Risk factors for progression of age-related macular degeneration: population-based Amish eye study. *J Clin Med*. 2022; 11: 5110. 36079043 10.3390/jcm11175110 PMC9457199

[28] Katschke KJ, Xi H, Cox C, et al. Classical and alternative complement activation on photoreceptor outer segments drives monocyte-dependent retinal atrophy. *Sci Rep*. 2018; 8(1): 7348. 29743491 10.1038/s41598-018-25557-8 PMC5943270

[29] Edwards MM, McLeod DS, Bhutto IA, Grebe R, Duffy M, Lutty GA. Subretinal glial membranes in eyes with geographic atrophy. *Invest Ophthalmol Vis Sci*. 2017; 58: 1352–1367. 28249091 10.1167/iovs.16-21229 PMC5358932

[30] Wu KHC, Madigan MC, Billson FA, Penfold PL. Differential expression of GFAP in early v late AMD: a quantitative analysis. *Br J Ophthalmol*. 2003; 87: 1159–1166. 12928288 10.1136/bjo.87.9.1159 PMC1771844

