# Supplementary material for: Systemic Sodium Iodate Injection as a Model for Expanding Geographic Atrophy
Source: Transl Vis Sci Technol. 2025 Jan 10;14(1):9. doi: 10.1167/tvst.14.1.9 (PMC11731155; doi:10.1167/tvst.14.1.9)
Supplement: Supplement 1 [file tvst-14-1-9_s001.docx]

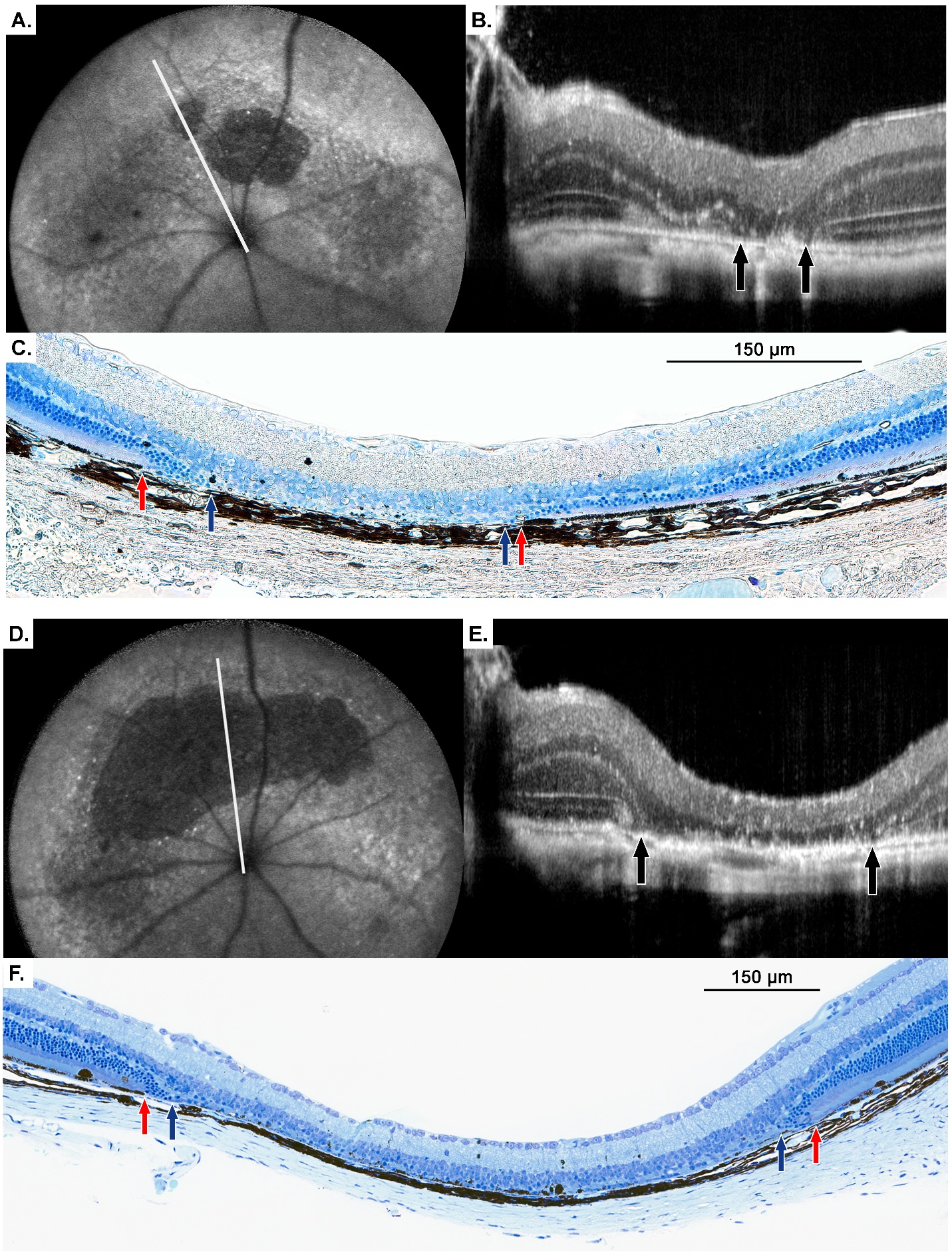


### Supplemental figure 1. Other examples of GA formation at 16 weeks.

(A,D) cSLO and (B,E) OCT images taken 16 weeks after injection of 20 mg/kg NaIO_3_. The white lines in the cSLO images correspond to where the OCT image was taken. (B,E) Black arrows represent cRORA borders. (C,F) Plastic section of the GA region stained with toluidine blue, with scale bar representing 150 µm. Red arrows show where the RPE layer ends, blue arrows show where photoreceptor nuclei end. A-C are from one mouse and D-F are from another.


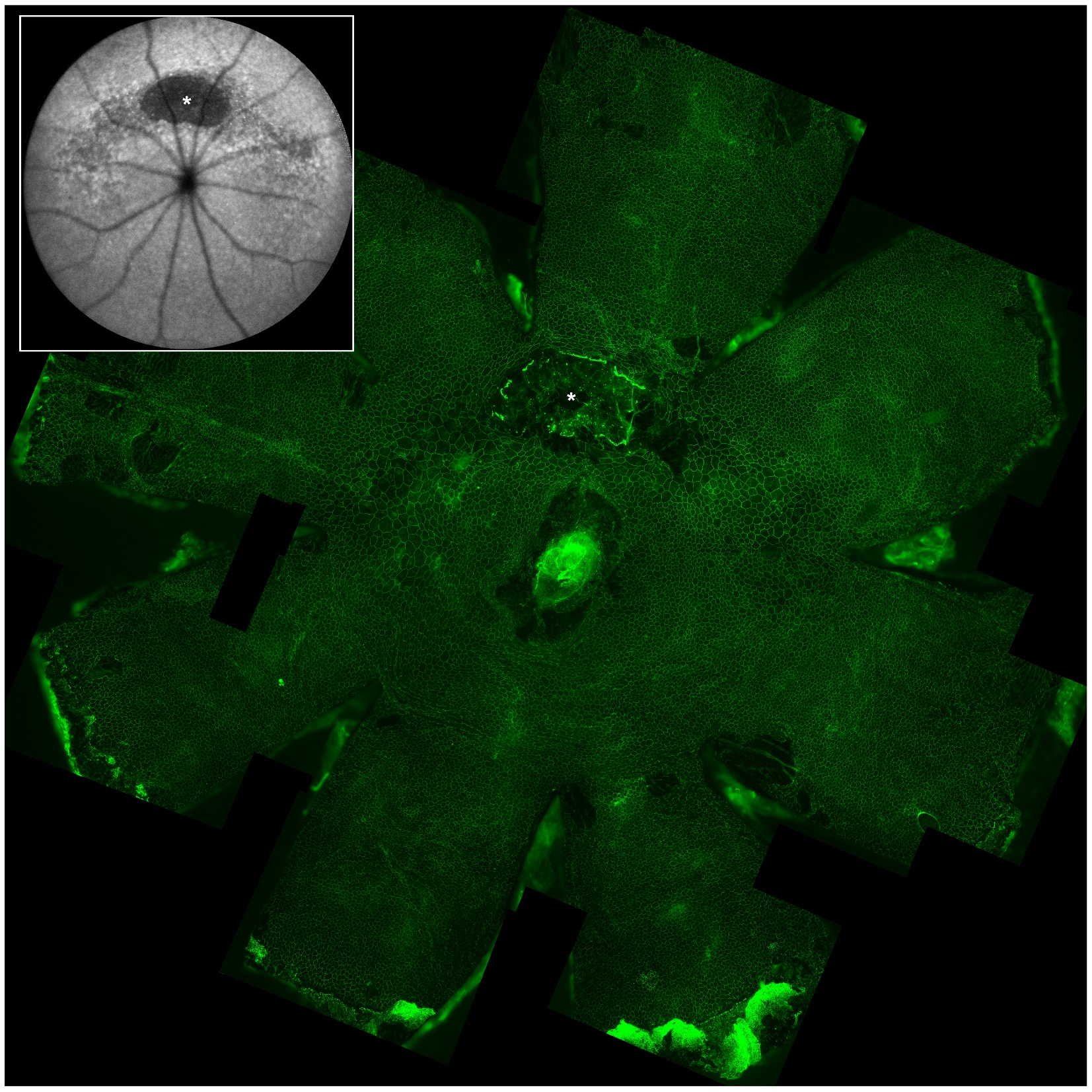


### Supplemental figure 2. Comparison of cSLO image to RPE flat mount.

RPE flat mount with ZO-1 staining (green) to mark border of RPE cells, compared with *in vivo* cSLO image taken shortly before the mouse was euthanized, 10 months post-injection. White asterisks represents the location of GA on both images.


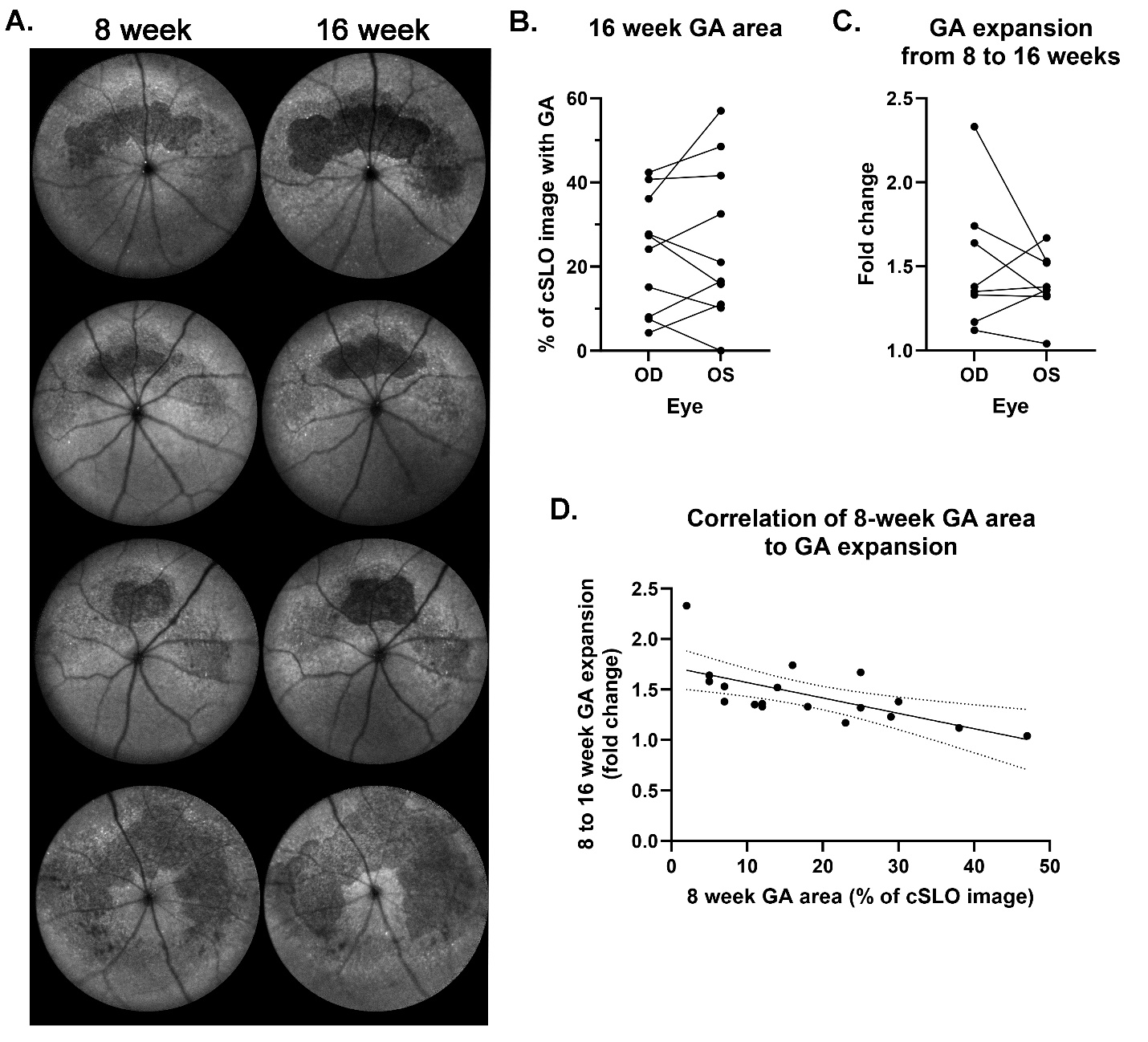


### Supplemental figure 3. Growth from 8 to 16 weeks and correlation between paired eyes.

(A) Example images from 8 weeks (left) and 16 weeks (right) after the 20 mg/kg NaIO_3_ injection, with GA in the superior retina. Each row of images is from the same eye. (B) Comparison of GA area and (C) expansion between OD and OS eyes, with lines connecting eyes from the same mouse (n=10 per group for B; n=8 per group for C). (B) Numbers are reported as the percentage of the 102° FOV retinal image occupied by GA. (D) Correlation between 8-week cRORA area and expansion from 8 to 16 weeks (n=18, R^2^=0.42). The bold line represents the line of best fit and the dotted lines indicate the 95% confidence bands around the best fit line. The slope (m=0.0045) is significantly non-zero (p<0.01). (B-D) Dots represent one eye.


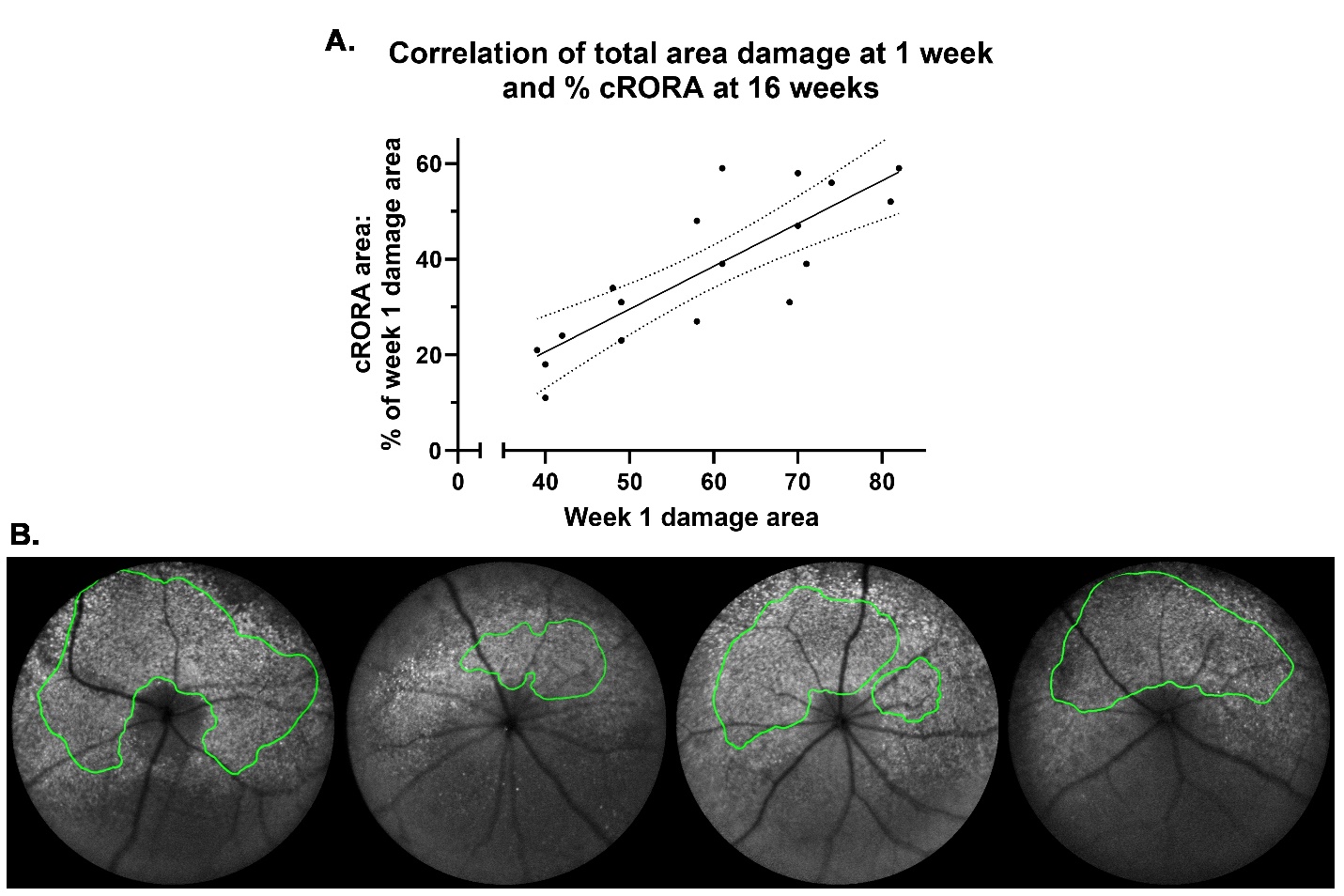


### Supplemental figure 4. Comparison of iRORA area at 1 week and cRORA area at 16 weeks.

(A) Correlation between one-week post-injection iRORA damage area and the percent of that damaged area becoming cRORA by 16 weeks post-injection (n=18, R^2^=0.68). Each dot represents one eye. The bold line represents the line of best fit and the dotted lines indicate the 95% confidence bands around the best fit line. The slope (m=0.895) is significantly non-zero (p<0.0001). (B) Example images taken one week after injection, with a green line overlay representing the borders of cRORA at 16 weeks.


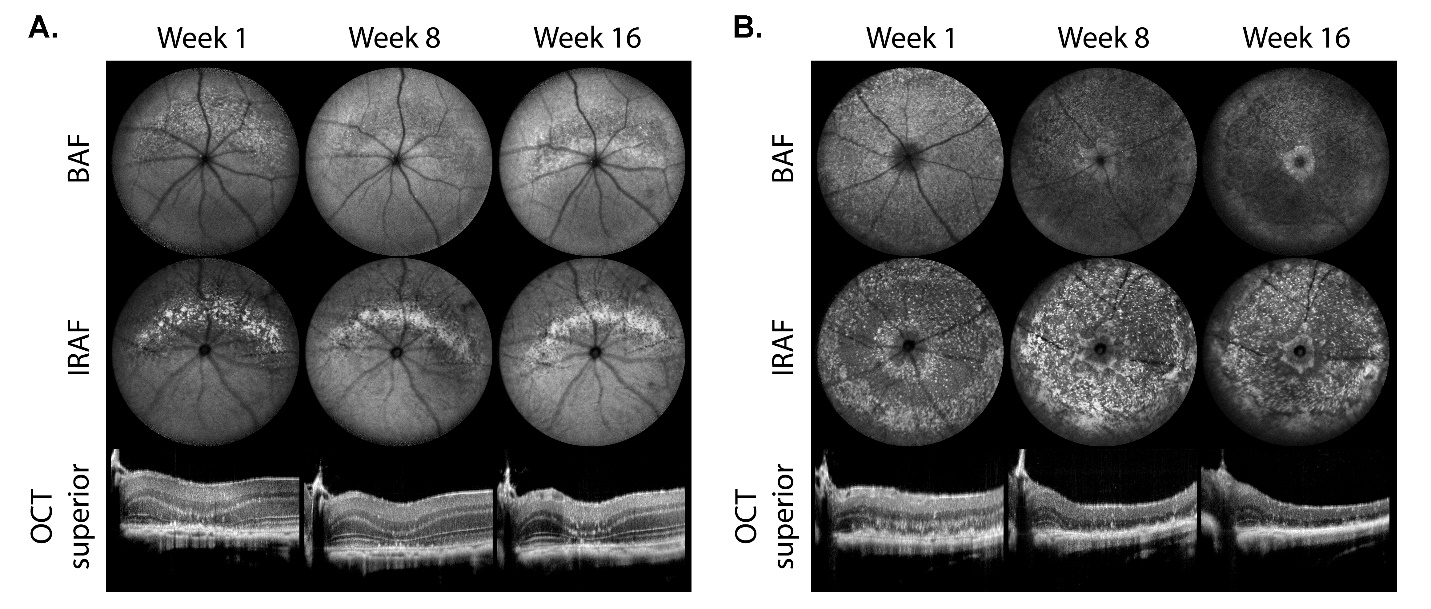


### Supplemental figure 5. Images of eyes unfit for GA expansion study.

Example images from a (A) minimally damaged eye and a (B) widespread damaged eye at 1, 8, and 16 weeks after the 20 mg/kg NaIO_3_ injection. The OCT images have the optic nerve at the left side, with the right side more superior. (A) Especially seen in the OCT image, the minimally damaged eye never developed cRORA and the RPE largely recovered by 16 weeks post injection. (B) The widespread damage had unclear GA border in the cSLO image at 8 weeks and has little room to see expansion in the 102° FOV image.


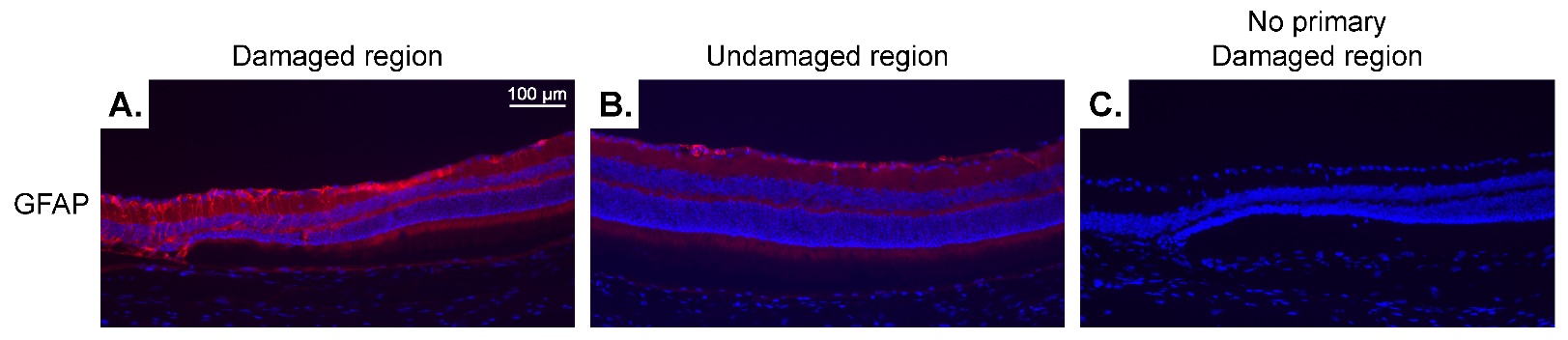


### Supplemental figure 6. IHC imaging of GFAP.

Representative images of glial fibrillary acidic protein (GFAP; red) at (A,C) the border of atrophy and (B) the undamaged region of the same retina. (A,C) cRORA is located on the far left side, with an iRORA region in the middle and an undamaged region on the right side. Scale bar in A is 100 µm and represents all images. Nuclei are labeled with DAPI (blue). GFAP expression was observed throughout the entire cRORA region (not shown).
